# Carbonyl Iron Particles’ Enhanced Coating Effect Improves Magnetorheological Fluid’s Dispersion Stability

**DOI:** 10.3390/ma17184449

**Published:** 2024-09-10

**Authors:** Fang Chen, Jie Zhang, Qinkui Guo, Yuchen Liu, Xiaobing Liu, Wenwu Ding, Shengnan Yan, Zhaoqiang Yan, Zhenggui Li

**Affiliations:** 1 Key Laboratory of Fluid and Power Machinery, Ministry of Education, Xihua University, Chengdu 610039, China; 15984174964@163.com (J.Z.); 19908119124@163.com (Q.G.); liuyuchenwalking@163.com (Y.L.); liuxb@mail.xhu.edu.cn (X.L.); wenwu_ding@sina.com (W.D.); yanshn@mail.xhu.edu.cn (S.Y.); 2 Zigong Zhaoqiang Sealing Products Industrial Co., Ltd., Zigong 643000, China; yanzhaoqiang12@126.com

**Keywords:** enhancement of coating effect, carbonyl iron particle, etching by hydrochloric acid, magnetorheological fluid, dispersion stability

## Abstract

The coating effect of 1,2-bis(triethoxysilyl)ethane (BTES) on carbonyl iron particles (CIPs) was enhanced by etching with hydrochloric acid (HCl) of various concentrations, and magnetorheological fluids (MRFs) with significantly improved dispersion stability were obtained. The microstructures, coating effect, and magnetism of CIPs were examined using scanning electron microscopy (SEM), automatic surface and porosity analysis (BTE), Fourier transform infrared spectroscopy (FTIR), thermogravimetric analysis (TGA), and a vibrating sample magnetometer (VSM), respectively. Furthermore, the rheological properties and dispersion stability of the MRFs were assessed by a rotating rheometer and a Turbiscan Tower. The results show that as the HCl concentration increased, nanopores appeared on CIPs and then disappeared, and the specific surface area of the particles increased and then decreased. When the concentration of HCl was 0.50 mol/L, the number of nanopores and the specific surface area of particles changed sharply. Not only that, the coated mass of BTES increased greatly and the saturation magnetization of particles decreased sharply. As the coated mass increased, without a magnetic field, the viscosity and shear stress of the MRFs increased, especially when the coated mass was more than 2.45 wt.%; while under a magnetic field, the viscosity and shear stress decreased, and the sedimentation rate of the MRFs decreased from 0.13 to 0.01 mm/h. By controlling the concentration of HCl for etching, the coating effect of CIPs was greatly enhanced, and thus an MRF with superior shear stress and excellent dispersion stability was obtained, which is significant in basic research and MRF-related applications.

## 1. Introduction

Magnetorheological fluid (MRF) is an intelligent material; its rheological properties change greatly with changes in the external magnetic field. MRFs are mainly composed of micron-scale magnetic particles, a carrier medium, and additives. Without a magnetic field, magnetic particles are randomly dispersed in the carrier medium, showing the characteristics of a freely flowing Newtonian fluid. Under a magnetic field, chain structures are arranged along the magnetic field direction, and the chain structures become thicker with an increase in the magnetic field’s strength, showing the characteristics of a solid-like pseudoplastic fluid [1,2,3]. Due to their special rheological properties, MRFs are widely used in buildings, cars, and seals, and in medical and other fields [4,5,6,7].

When particles in an MRF are unevenly dispersed or aggregated, its rheological properties are affected, causing problems such as a decrease in response speed, unstable performance, and shortened service life. Micron-sized carbonyl iron particles (CIPs) have high saturation magnetization, excellent paramagnetic characteristics, optimal size, and easy accessibility. Thus, they are typically utilized as magnetic particles in MRFs. However, the most severe MRF problems are sedimentation and low yield stress, which are attributed to the obvious density difference between CIPs (7.8 g/cm^3^) and the carrier medium (e.g., silicone oil at 0.963 g/cm^3^) and the large surface energy of particles [8,9]. Thus, a number of strategies have been used to reduce the sedimentation of MRFs, such as the use of rod-shaped magnetic particles [10], the addition of graphene oxide [11], the addition of fumed silica [12], the use of high-viscosity fluid [13,14], and coating particles with polymer [15,16].

Among these, coating particles with polymers has proven to be effective. For example, after being coated with polymer polystyrene foam, the sedimentation rate of the MRF decreased from 80% to 30% [17]. After CIPs were coated with poly(methyl methacrylate) (PMMA) by emulsion polymerization, the sedimentation rate of the MRF decreased from 80% to 70% [18]. After CIPs were coated with tragacanth gum, the sedimentation rate of the MRF decreased from 15% to 10% [19].

To further enhance the effect of coating particles with polymers, Belyavskii et al. indicated that short chain molecules could promote their graft density on the particles [20]. Moreover, the number of active functional groups of polymers also affects the coating effect. The coating effect of “bis-silane” is better than that of “mono-silanes” [21]. In addition, it was reported that an increase in the -OH functional groups on CIPs could enhance their surface activity, and the surfaces of CIPs showed Lewis acidity after they were etched with HCl [22]. Moreover, an increase in the specific surface area of particles could also improve the coated mass of polymers [23]. It follows that the chain lengths of silane coupling agents, the number of grafted functional groups, the number of -OH functional groups, and the specific surface area of particles all affect the coating effect of CIPs and the dispersion stability of MRFs. 

The dispersion stability of the obtained MRF still cannot meet long-term stable application requirements and needs to be further improved. In this study, CIPs were etched with HCl of different concentrations to increase the coated mass of BTES on CIPs. The particles were dispersed into polyalphaolefin synthetic oil (PAO) to prepare the MRFs. The microscopic morphology, coating effect, and magnetic properties of etched and coated CIPs, as well as the rheological properties and dispersion stabilities of the MRFs, were characterized. An MRF with high yield stress and high dispersion stability was obtained, which will promote MRFs’ application effects in, and is significant for, basic theoretical research.

## 2. Materials and Methods

The parameters of materials used in this experiment are shown in Table 1.

**Figure 1:**
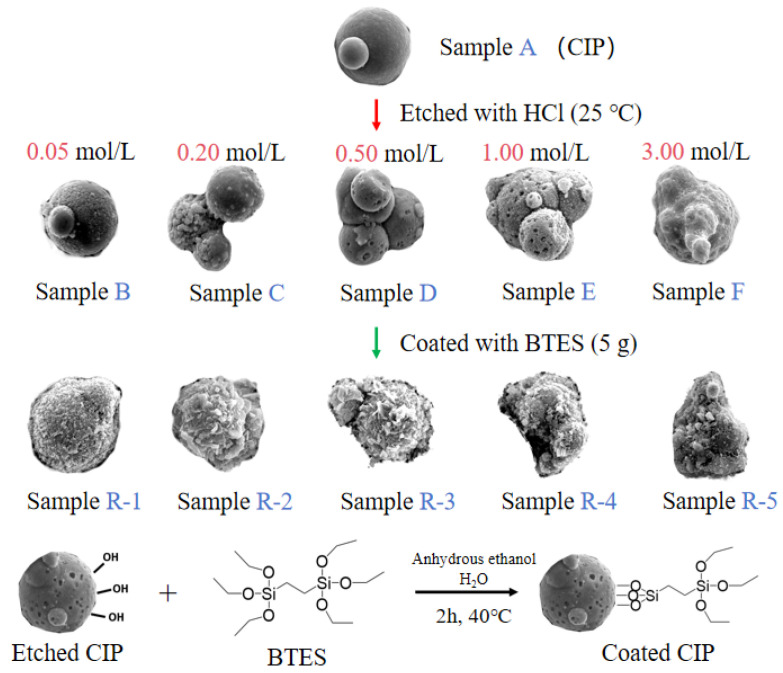
The etching and coating process of particles.

Particles preparation: Figure 1 shows the etching and coating process of particles. Firstly, 40 g of CIPs was etched by HCl solutions with different concentrations at 250 rpm for 10 min to obtain etched particles, labelled as samples A to F. Secondly, etched particles were coated with 5 g of BTES in a mixed solution of 200 mL of ethanol and 15 mL of water at 250 rpm for 2 h. Finally, coated particles were obtained and labelled as samples R-1 to R-5, as shown in Table 2.

Preparation of MRFs: MRFs were prepared by mixing the coated particles and 60 wt.% of PAO at 600 rpm for 2 h, and were labeled as M-0 to M-6, as shown in Table 2.

The microscopic morphology of CIPs was characterized using scanning electron microscopy (SEM, Phenom Pharos G2, Phenom, Eindhoven, The Netherlands), and the magnification was 20,000×. The specific surface area and porous structure characteristics of CIPs were tested by an automatic surface and porosity analyzer (BTE, Micromeritics ASAP 2460, Micromeritics, GA, USA) at 120 °C by nitrogen adsorption. The coated effect of particles was studied by Fourier transform infrared spectroscopy (FTIR, Nicolet iS 10, Thermo Fisher, USA), and the wavenumber range was from 500 to 4000 cm^−1^. The coated mass of the particles was tested by a thermal gravimetric analyzer (TGA, Setaram Labsys Evo, Setaram, Lyon, France). Under the condition of a nitrogen atmosphere, the temperature range was from 30 °C to 600 °C, and the heating rate was 20 °C/min. The magnetic properties of the particles were measured by a vibrating sample magnetometer (VSM, Lake Shore 7404, Lake Shore, Westerville, OH, USA), and the applied field range was from −2.0 × 10^4^ Oe to +2.0 × 10^4^ Oe at 25 °C. The magnetorheological properties of the MRFs were measured using a rotary rheometer (MCR 302e Anton Paar, Anton Paar, Graz, Austria). The measuring system was a PP25-SN3320, the test unit was a P-PTD200 (Anton Paar, Graz, Austria), and the magnetic field was controlled by the magnetic control system (MRD 170/1T, Anton Paar, Graz, Austria). The sample platform was a parallel plate with a shear disc gap of 1 mm. The range of the magnetic field strength was from 0 kA/m to 174 kA/m, and the shear rate ranged from 0.1 s^−1^ to 1000 s^−1^. The dispersion stability of the MRFs were measured by a Turbiscan Tower (Turbiscan-lab, Formulaction, Toulouse, France). The MRFs were placed in cylindrical glass tubes and periodically scanned from bottom to top by a beam of near-infrared light (*λ* = 880 nm), and the migration rates of the particles were derived. Experimental results were repeated at least three times.

## 3. Results

### 3.1. The Morphology of the Particles

**Figure 2:**
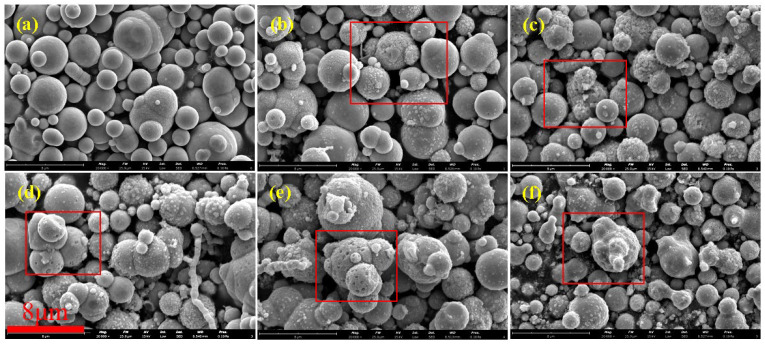
SEM images of particles etched by HCl with different concentrations: (**a**) 0 mol/L, (**b**) 0.05 mol/L, (**c**) 0.20 mol/L, (**d**) 0.50 mol/L, (**e**) 1.00 mol/L, and (**f**) 3.00 mol/L.

Figure 2 shows SEM images of CIPs etched by HCl with different concentrations. Figure 2a shows that unetched CIPs were a regular spherical shape and their surface was smooth. Figure 2b shows that spot-like solids appeared on CIPs and their surface was rough. This is due to the fact that after the oxide covering of the component CIPs was removed by HCl, α-Fe nanoparticles were exposed [24]. Figure 2c shows that more spot-like solids appeared on CIPs, and it is worth noting that nanopores also appeared. Figure 2d shows that more nanopores appeared on CIPs. Figure 2e shows that the spot-like solids disappeared from CIPs. Figure 2f shows that nanopores grew into depressions on CIPs. With an increase in the HCl concentration, the number of nanopores on the particles increased gradually, and eventually disappeared when the concentration was 3.00 mol/L. During the etching process, a large number of Fe-OH groups generated on the surfaces of the particles. The particles’ surfaces showed Lewis acidity after being etched with HCl, resulting in the chemisorption of -OH from the H_2_O on the etched particles’ surfaces, which promoted the coating effect of the silane coupling agent on CIPs and prevented the further oxidization of coated particles [25].

Table 3 shows the parameters of CIPs etched by HCl at different concentrations. In the table, *S*_BET_ represents the specific surface area of CIPs, *S*_BJH_ denotes the specific surface area of nanopores, and *V*_BJH_ indicates the specific volume of nanopores. After being etched by 0.05 mol/L HCl, the *S*_BET_ increased from 0.4640 m^2^/g to 0.5045 m^2^/g. However, changes in the *S*_BJH_ and *V*_BJH_ were not significant. As the HCl concentration increased, the *S*_BET_, *S*_BJH_, and *V*_BJH_ all exhibited significant increases. When the HCl concentration reached 1.00 mol/L, the *S*_BET_ increased to 43.5450 m^2^/g, while the *S*_BJH_ and *V*_BJH_ increased to 34.6350 m^2^/g and 0.033943 cm^3^/g, respectively. Nevertheless, when the HCl concentration reached 3 mol/L, the *S*_BET_, *S*_BJH_, and *V*_BJH_ decreased significantly, indicating that the porous structures of the particles disappeared.

**Figure 3:**
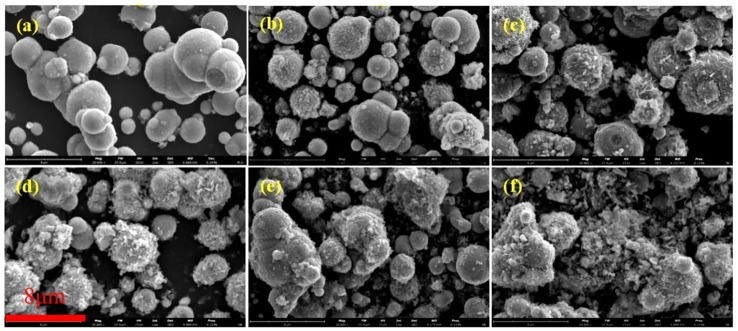
SEM images of coated CIPs etched by HCl of different concentrations: (**a**) 0 mol/L, (**b**) 0.05 mol/L, (**c**) 0.20 mol/L, (**d**) 0.50 mol/L, (**e**) 1.00 mol/L, and (**f**) 3.00 mol/L.

Figure 3 depicts SEM images of coated CIPs etched by HCl at different concentrations. As observed from these images, with increases in HCl concentration, the coated BTES on the particles gradually increased, suggesting that the etching process can enhance the coated mass [26].

### 3.2. The Coating Effect of BTES on Particles

**Figure 4:**
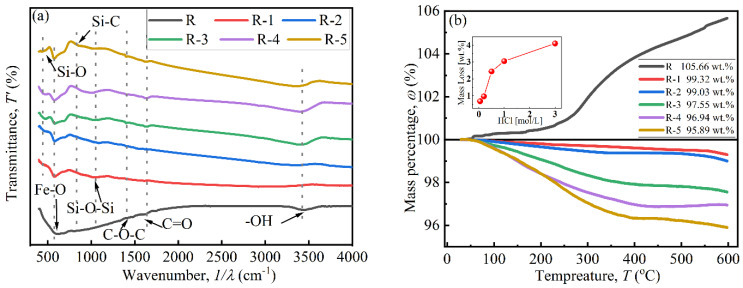
The coating effect of CIPs: (**a**) FTIR curves and (**b**) TGA curves.

The coating effect of BTES on particles was investigated using FTIR and TGA curves, as shown in Figure 4. In Figure 4a, peaks located at 630.69cm^−1^, 1357.81 cm^−1^, and 1384.81 cm^−1^ indicate the existence of Fe-O, C-O-C, and C=O bonds, respectively. The peak at 3446.60 cm^−1^ indicates the presence of an -OH bond, attributed to attached water on the particles [27,28]. Peaks at 441.67 cm^−1^, 894.92 cm^−1^, and 995.21 cm^−1^ in the curves of coated particles confirm the existence of Si-O, Si-C, and Si-O-Si bonds, respectively, which were derived from BTES. This indicates that a polycondensation reaction occurred between the hydrolytic functional groups (Si-OH) [26].

In Figure 4b, a mass increase only appears in curve R near 300 °C, which was attributed to the chemical reaction between N_2_ from the testing environment and uncoated CIP particles [29]. The mass of coated particles decreased with a rise in temperature due to the decomposition of the coated BTES. The decrease began at about 100 °C, which was attributed to adsorbed water [29]. The coated mass increased with increases in HCl concentration, which were 0.68 wt.%, 0.97 wt.%, 2.45 wt.%, 3.06 wt.%, and 4.11 wt.%. When the HCl concentration was below 0.20 mol/L, the coated mass was less than 1 wt.%. When the concentration was above 0.50 mol/L, the coated mass greatly increased to 2.45 wt.%, indicating that it is difficult to coat BTES onto CIPs when the HCl concentration is insufficient [16]. Combined with SEM images in Figure 3, the increase in coated mass was due to the sharp increase in the specific surface area and -OH functional groups of particles after being etched by HCl, especially when the concentration was high enough and reached 0.5 mol/L [30].

**Figure 5:**
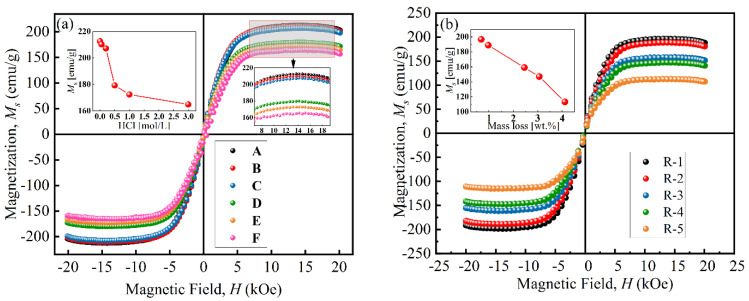
Magnetization curves of (**a**) uncoated particles and (**b**) coated particles.

Figure 5 presents the magnetization curves of particles at room temperature. The VSM curves of all particles are close to an “S” shape, and their coercive force was small, indicating that the particles had similar properties to superparamagnetic materials. As shown in Figure 5a, the saturation magnetization (*M*_s_) of particles decreased from 212.7 emu/g to 164.8 emu/g with an increase in HCl concentration, which was attributed to the chemical reaction [26]. When the concentration was above 0.50 mol/L, the *M*_s_ sharply decreased to 179.67 emu/g due to the formation of a porous structure during the etching process [31]. Figure 5b shows that the *M*_s_ of coated particles decreased from 196.7 emu/g to 113.3 emu/g with increases in coated mass. This was because the existence of non-magnetic BTES increased the distance between magnetic cores and reduced the magnetic interaction force between particles [17].

### 3.3. Rheological Properties of MRFs

**Figure 6:**
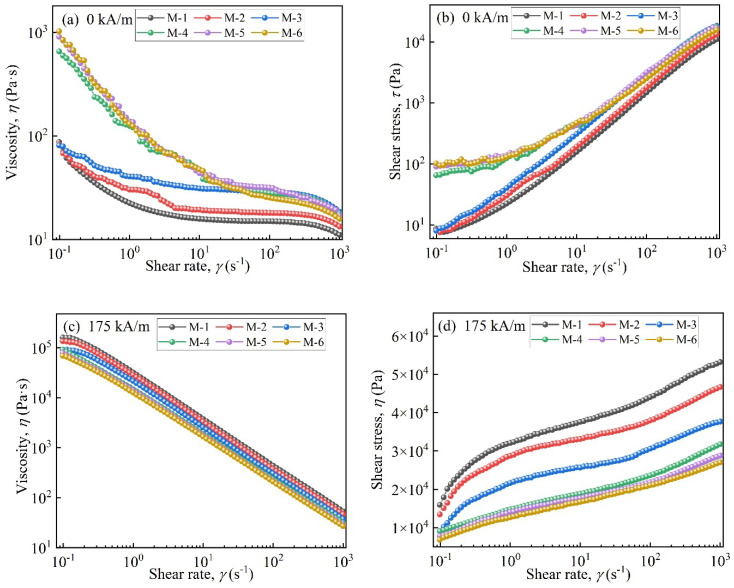
Rheological properties of the MRFs: (**a**) viscosity vs. shear rate (0 kA/m), (**b**) shear stress vs. shear rate (0 kA/m), (**c**) viscosity vs. shear rate (175 kA/m), and (**d**) shear stress vs. shear rate (175 kA/m).

Figure 6 displays the rheological properties of MRFs. Figure 6a shows that in the absence of a magnetic field, the viscosity of MRFs decreased as the shear rate increased. This was attributed to the disentanglement and straightening of entangled molecules and the destruction of the three-dimensional network structure [32]. When the coated mass was above 2.45 wt.%, the viscosity increased by an order of magnitude at the beginning of shearing. A faster downward trend appeared compared to M-1, M-2, and M-3. This was attributed to the enhanced entanglement effect between coated particles and carrier medium molecules and the formation of stronger three-dimensional network structures [33]. Additionally, at the beginning of shearing, the shear stresses of M-4, M-5, and M-6 were significantly higher than those of M-1, M-2, and M-3, with coated masses less than 2.45 wt.%.

In Figure 6c,d, under a magnetic field, with an increase in coated mass the viscosity and shear stress of MRFs decreased. This was lower than those of MRFs prepared by uncoated CIP particles. This was due to the formation of porous structures on CIPs after they were etched with HCl and the coated non-magnetic layer [34].

### 3.4. The Dispersion Stability of the MRFs

**Figure 7:**
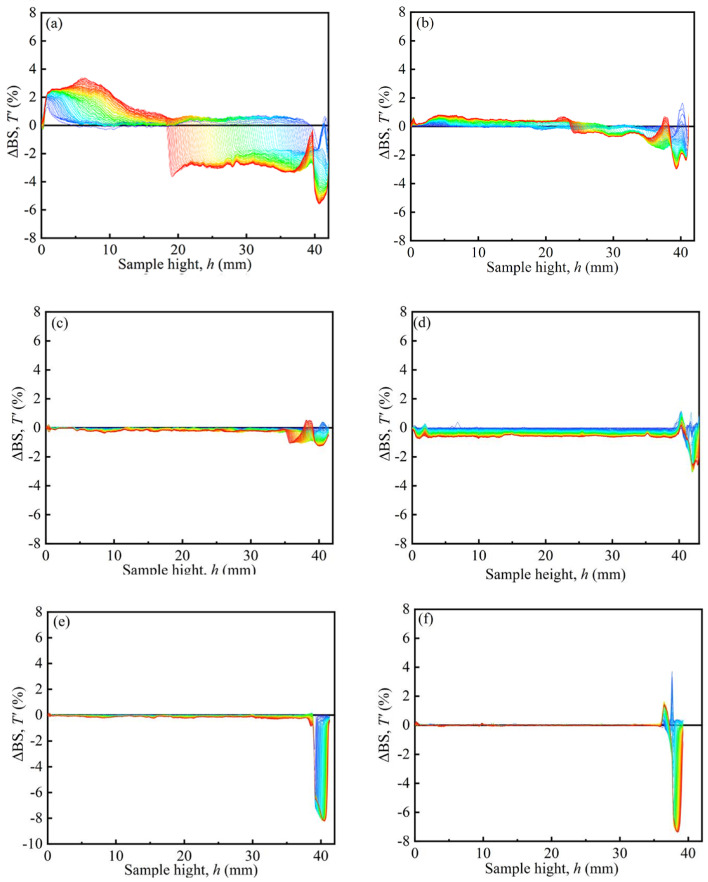
Typical Turbiscan spectra of the MRFs: (**a**) M-1, (**b**) M-2, (**c**) M-3, (**d**) M-4, (**e**) M-5, and (**f**) M-6.

Figure 7 shows the typical Turbiscan spectra of the MRFs. The dispersion stability of MRFs was tested by a Turbiscan Tower. The samples of MRFs were placed in sample units with a height of 40 mm. Scans were repeated within 7 days. The results are denoted by the colors of the curves, and ΔBS represents the backscattered intensity. The peak thickness was from 40 mm to the turning point between the positive value and the negative value of ΔBS, expressed as the sedimentation condition of the MRFs [35]. In Figure 7, it can be seen that the peak thickness gradually decreased with an increase in coated mass on particles, indicating that the sedimentation of MRFs gradually decreased.

**Figure 8:**
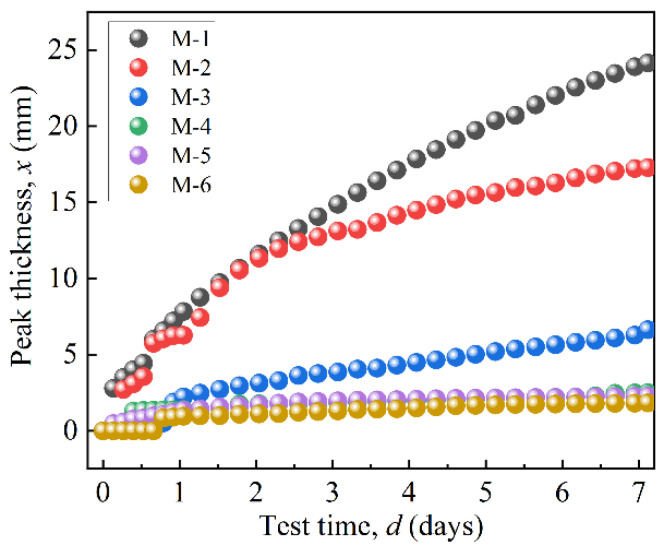
The peak thicknesses variation in particles over 7 days.

Figure 8 shows the peak thicknesses of the MRFs over a period of 7 days. Figure 8’s results were obtained based on the variation in curves in Figure 7. ΔBS of M-1 changes from a negative peak to a positive peak at 15.85 mm, indicating the sedimentation position. Its peak thickness was obtained as 24.15 mm, indicating the sedimentation amount of MRF. The peak thickness of M-2 was 17.28 mm, and its turning point was 12.72 mm. The peak thickness of M-2 was smaller than that of M-1, indicating that the coated BTES had a positive effect on the dispersion stability of the MRFs. Furthermore, with an increase in coated mass, the dispersion stability of the MRFs greatly improved.

Table 4 shows the peak thicknesses and sedimentation rates of the MRFs after 7 days of sedimentation. As the coated mass increased, the peak thickness decreased from 17.28 mm to 1.83 mm, and the sedimentation rate of the MRFs decreased from 0.13 mm/h to 0.01 mm/h. This was due to the formation of stronger three-dimensional network structures, which enhanced the viscous resistance of the particles [32]. In addition, when the coated mass was higher than 2.45 wt.%, the sedimentation rate was close to 0.01 mm/h, indicating that a further increase in coated mass had little effect on the dispersion stability of the MRFs. Meanwhile, the excessive etching of CIPs caused the saturation magnetization of particles to reduce, thereby seriously reducing the shear stress of the MRFs under a magnetic field.

When CIPs were etched by 0.5 mol/L HCl, the coated mass loss was 2.45 wt.%, and the sedimentation rate of the MRF was 0.01 mm/h. Moreover, the decrease in field-induced shear stress caused by the reduction in the saturation magnetization of the particles was small.

## 4. Conclusions

CIPs were etched by HCl with different concentrations and then coated with BTES and dispersed into PAO to prepare MRFs. The microstructures, coating effects, and magnetism of the particles, as well as the rheological properties and dispersion stability of the MRFs, were systematically characterized. As the HCl concentration increased from 0.05 mol/L to 3.00 mol/L, the number of nanopores increased and eventually disappeared. Simultaneously, the specific surface area of the particles increased and then decreased. When the HCl concentration was 0.50 mol/L, the number of nanopores and the specific surface area of particles changed sharply. Furthermore, the coated mass sharply increased to 2.45 wt.% and the saturation magnetization sharply decreased due to the formation of porous structures and the high specific surface area of the particles, caused by etching by highly active H^+^ from HCl. As the coated mass increased, the viscosity and shear stress of the MRFs decreased under the magnetic field, and the sedimentation rate of particles decreased. When the coated mass was above 2.45 wt.%, compared with MRFs prepared by uncoated particles, the viscosity and shear stress increased by an order of magnitude at the beginning of shearing, and the sedimentation rate of particles in MRFs was close to 0.01 mm/h. The obtained MRFs struck a balance between coating effects, magnetic properties, and overall performance under a magnetic field.

## Figures and Tables

**Table 1:** The parameters of materials used in this experiment.

Materials	Chemical Formula	Purity	Manufacturer
Hydrochloric acid	HCl	AR	Chengdu Colon Chemical Co., Ltd. (Chengdu, China)
Anhydrous ethanol	C_2_H_5_OH	AR	
Carbonyl iron particles	Fe(CO)_5_	≥99.9%	Shanghai Xiangtian Nanomaterials Co., Ltd. (Shanghai, China)
1,2-bis(triethoxysilyl)ethane	C_14_H_34_O_6_Si_2_	≥95%	Shanghai Maclin Technology Co., Ltd. (Shanghai, China)
Polyalphaolefin synthetic oil	PAO	≥99%	Shanghai Nac Lubrication Technology Co., Ltd. (Shanghai, China)

**Table 2:** Parameters of prepared MRFs.

The Concentration of HCl	Uncoated CIPs	Coated CIPs	MRF	Carrier Liquid (8 g)
0 mol/L	A	R-0	M-0	PAO-100
0.05 mol/L	B	R-1	M-1	
0.20 mol/L	C	R-2	M-2	
0.50 mol/L	D	R-3	M-3	
1.00 mol/L	E	R-4	M-4	
3.00 mol/L	F	R-5	M-5	

**Table 3:** Parameters of particles etched by HCl with different concentrations.

Sample	*S*_BET_ (m^2^/g)	*S*_BJH_ (m^2^/g)	*V*_BJH_ (cm^3^/g)
A	0.4640	0.4400	0.000953
B	0.5045	0.4326	0.001902
C	2.6053	1.6847	0.003522
D	37.7697	27.0089	0.026672
E	43.5450	34.6350	0.033943
F	0.9172	1.1214	0.004098

**Table 4:** Migration rates of particles after sedimentation for 7 days.

Samples	Peak Thickness (mm)	Sedimentation Rate (mm h^−1^)
M-1	24.15	0.13
M-2	17.28	0.09
M-3	6.64	0.04
M-4	2.50	0.01
M-5	2.28	0.01
M-6	1.83	0.01

## Data Availability

The original contributions presented in the study are included in the article, further inquiries can be directed to the corresponding author.

## References

[1] Cvek M., Mrlik M., Ilcikova M., Plachy T., Sedlacik M., Mosnacek J., Pavlinek V. (2015). A facile controllable coating of carbonyl iron particles with poly (glycidyl methacrylate): A tool for adjusting MR response and stability properties. J. Mater. Chem. C..

[2] Feijoo A.V., Lopez-Lopez M.T., Galindo-Gonzalez C., Stange S., Nguyen T.T., Mammeri F., Merash S., Ponton A. (2020). Rheological investigation of magnetic sensitive biopolymer composites: Effect of the ligand grafting of magnetic nanoparticles. Rheol. Acta.

[3] Marins J.A., Plachý T., Kuzhir P. (2019). Iron-sepiolite magnetorheological fluids with improved performances. J. Rheol..

[4] Kordonski W.I., Jacobs S.D. (1996). Magnetorheological finishing. Int. J. Mod. Phys. B.

[5] Miriyala D.N., Goyal P.S. (2022). Effect of MagneticField on the Damping Behavior of a Ferrofluid based Damper. arXiv.

[6] Chen F., Zhang J., Li Z., Yan S., Li W., Yan Z., Liu X. (2024). Effect of the surface coating of carbonyl iron particles on the dispersion stability of magnetorheological fluid. Sci. Rep..

[7] Scherer C., Neto A.M.F. (2005). Ferrofluids: Properties and applications. Braz. J. Phys..

[8] Kim M.S., Liu Y.D., Park B.J., You C.Y., Choi H.J. (2012). Carbonyl iron particles dispersed in a polymer solution and their rheological characteristics under applied magnetic field. J. Ind. Eng. Chem..

[9] Sun Y., Wang Y., Deng H., Sang M., Gong X. (2022). Effect of MXene nanosheets attached to carbonyl iron microspheres on the performance and stability of magnetorheological fluid. J. Ind. Eng. Chem..

[10] Bell R.C., Karli J.O., Vavreck A.N., Zimmerman D.T., Ngatu G.T., Wereley N.M. (2008). Magnetorheology of submicron diameter iron microwires dispersed in silicone oil. Smart Mater. Struct..

[11] Zhang W.L., Kim S.D., Choi H.J. (2013). Effect of graphene oxide on carbonyl-iron-based magnetorheological fluid. IEEE Trans. Magn..

[12] Aruna M.N., Rahman M.R., Joladarashi S., Kumar H., Bhat P.D. (2021). Influence of different fumed silica as thixotropic additive on carbonyl particles magnetorheological fluids for sedimentation effects. J. Magn. Magn. Mater..

[13] Xie L., Choi Y.T., Liao C.R., Wereley N.M. (2016). Long term stability of magnetorheological fluids using high viscosity linear polysiloxane carrier fluids. Smart Mater. Struct..

[14] Kumar S., Sehgal R., Wani M.F., Sharma M.D. (2021). Stabilization and tribological properties of magnetorheological (MR) fluids: A review. J. Magn. Magn. Mater..

[15] Lee J.W., Hong K.P., Kwon S.H., Choi H.J., Cho M.W. (2017). Suspension rheology and magnetorheological finishing characteristics of biopolymer-coated carbonyliron particles. Indus. Eng. Chem. Res..

[16] Cheng H., Wang M., Liu C., Wereley N.M. (2018). Improving sedimentation stability of magnetorheological fluids using an organic molecular particle coating. Smart Mater. Struct..

[17] Chuah W.H., Zhang W.L., Choi H.J., Seo Y. (2015). Magnetorheology of core–shell structured carbonyl iron/polystyrene foam microparticles suspension with enhanced stability. Macromolecules.

[18] Jiang W., Zhu H., Guo C., Li J., Xue Q., Feng J., Gong X. (2010). Poly (methyl methacrylate)-coated carbonyl iron particles and their magnetorheological characteristics. Polym. Int..

[19] Swaroop K.V., Aruna M.N., Kumar H., Rahman M.R. (2020). Rheological characterization of tragacanth gum coated carbonyl particles based magnetorheological fluid, Advances in mechanical design, materials and manufacture: Proceeding of the Second International Conference on Design. Mater. Manuf..

[20] Belyavskii S.G., Mingalyov P.G., Giulieri F., Combarrieau R., Lisichkin G.V. (2006). Chemical modification of the surface of a carbonyl iron powder. Prot. Met..

[21] Van Ooij W.J., Zhu D.Q., Prasad G., Jayaseelan S., Fu Y., Teredesai N. (2000). Silane based chromate replacements for corrosion control, paint adhesion, and rubber bonding. Surf. Eng..

[22] Joseph Y., Ranke W., Weiss W. (2000). Water on FeO(111) and Fe_3_O_4_(111): Adsorption Behavior on Different Surface Terminations. J. Phys. Chem. B.

[23] Merkel M.P., Dimonie V.L., El-Aasser M.S., Vanderhoff J.W. (1987). Process parameters and their effect on grafting reactions in core/shell latexes. J. Polym. Sci. Pol. Chem..

[24] König R., Müller S., Dinnebier R.E., Hinrichsen B., Müller P., Ribbens A., Pistidda C. (2017). The crystal structures of carbonyl iron powder–revised using in situ synchrotron XRPD. Z Krist. Cryst. Mater..

[25] Jafarian M., Afghahi S.S.S., Atassi Y., Salehi M. (2018). Enhanced microwave absorption characteristics of nanocomposite based on hollow carbonyl iron microspheres and polyaniline decorated with MWCNTs. J. Magn. Magn. Mater..

[26] Aziz S.A.B.A., Mazlan S.A., Nordin N.A., Rahman N.A.N.A., Ubaidillah U., Choi S.B., Mohamad N. (2019). Material characterization of magnetorheological elastomers with corroded carbonyl iron particles: Morphological images and field-dependent viscoelastic properties. Int. J. Mol. Sci..

[27] Chen Y., Chen H.R., Shi J.L. (2014). Construction of homogenous/heterogeneous hollow mesoporous silica nanostructures by silica-etching chemistry: Principles, synthesis, and applications. Acc. Chem. Res..

[28] Swaroop K.V., Aruna M.N., Kumar H., Rahman M.R. (2021). Investigation of steady state rheological properties and sedimentation of coated and pure carbonyl iron particles based magneto-rheological fluids. Mater. Today.

[29] Yang K., Gu M. (2010). Enhanced thermal conductivity of epoxy nanocomposites filled with hybrid filler system of triethylenetetramine-functionalized multi-walled carbon nanotube/silane-modified nano-sized silicon carbide. Compos. Part A Appl. Sci. Manuf..

[30] Yin C., Cao Y., Fan J., Bai L., Ding F., Yuan F. (2013). Synthesis of hollow carbonyl iron microspheres via pitting corrosion method and their microwave absorption properties. Appl. Surf. Sci..

[31] Burhannuddin N.L., Nordin N.A., Mazlan S.A., Aziz S.A.A., Kuwano N., Jamari S.K.M. (2021). Physicochemical characterization and rheological properties of magnetic elastomers containing different shapes of corroded carbonyl iron particles. Sci. Rep..

[32] Zhao P., Du T., Dong N.M.X., Qi M. (2023). Effect of interfacial shear strength between magnetic particles and carrier liquid on rheological properties of magnetorheological fluids. J. Mol. Liq..

[33] Gorodov V.V., Kostrov S.A., Kamyshinskii R.A., Kramarenko E.Y., Muzafarov A.M. (2018). Modification of carbonyl iron particles by carboxyl-containing polydimethylsiloxanes. Russ. Chem. Bull..

[34] Plachy T., Cvek M., Munster L., Hanulikova B., Suly P., Vesel A., Cheng Q. (2021). Enhanced magnetorheological effect of suspensions based on carbonyl iron particles coated with poly(amidoamine) dendrons. Rheol. Acta.

[35] Cvek M., Mrlik M., Moucka R., Sedlacik M. (2018). A systematical study of the overall influence of carbon allotrope additives on performance, stability and redispersibility of magnetorheological fluids. Colloid Surf. A.

